# Neuropathic Pains in the Age of Post-COVID-19-Vaccination

**DOI:** 10.1192/j.eurpsy.2025.461

**Published:** 2025-08-26

**Authors:** Y. I. Cao

**Affiliations:** Founder, Scientifique Global Limited, Hong Kong, Hong Kong

## Abstract

**Introduction:**

Mainstream literature classifies SARS-CoV-2 positive-sensed single-stranded RNA (ssRNA), and only a few literature mentioned the method being Reverse Transcription–Polymerase Chain Reaction -- one limited to ssRNA studies without method improvement of RNA interference (Wang *et al.* BMC Bio 2018; 18). Studies found the 3’-to-5’ exoribonuclease activity within CoV nonstructural protein 14 (NSP14) critical for CoV high-fidelity replication (Smith & Denison, PLoS Pathog 2013; 9 e1003760), and NSP15 a distinctive endoribonuclease able to cleave both ss- and double-stranded RNA (dsRNA) effectively (Frazier *et al.* NA Res 2022; 50 8290-8301). While MERS-CoV inhibit oligoadenylate synthetase–ribonuclease L, protein kinase R, and interferon (IFN), CoV-2 activates the former two and induces minimal levels of IFN (Li *et al.* PNAS 2021; 118 e2022643118), corroborating with S2 protein’s homogeneity with HIV gp41 (Zhang & Yap JMS: THEOCHEM 2004; 677 73-76) with differentiated impacts on macrophage activities via interleukin 6 (Ascierto *et al.* JIC 2021; 9). **Image 1** indicates the post-vaccination pericarditis is caused by negative charge interference during depolarization in NCT05711810.

**Objectives:**

Primary objective of advancing treatment designs followed the fixed effect metaanalysis model and gathered relevant data (Nikolakopoulou *et al.* EBMH 2014; 17 64). Secondary objective is to compare effects between presynaptic and postsynaptic treatment efficacies in order to determine infection depth for post-COVID-19-vaccination neuropathic pain to appear, and adverse events (AEs) are collected for random effects metaanalysis. Tertiary objective is to weigh the evidences whether COVID-19 is ssRNA or dsRNA.

**Methods:**

With the framework and paradigm of sebaceous immunobiology, the pathway bypassing blood-brain barrier is found with steroidogenesis (Pachankis JP 2023; 26 615; Pachankis GJMR 2023; 23C 5-11). NCT05839236 and NCT06357104 trials’ metaanalysis are illustrated in **image 2** with the observational protocol NCT06107348 in **image 3**.

**Results:**

Sebaceous (purpura and ecchymoses) AEs appeared with postsynaptic treatments with valproate, and comparatively, presynaptic treatments with gabapentin afterwards attenuated them. Presynaptic treatments of gabapentin shows superiority by the equivalence tests on neuropathic pains’ attenuation in duration and intensities. The considerations of Ca^2+^ channel inhibition by the adoption of gabapentin in the proton-coupled electron transport chain are consistent with the electrocardiogram indications of negative charge interference on cardiac activities.

**Image 1:**

**Figure:**
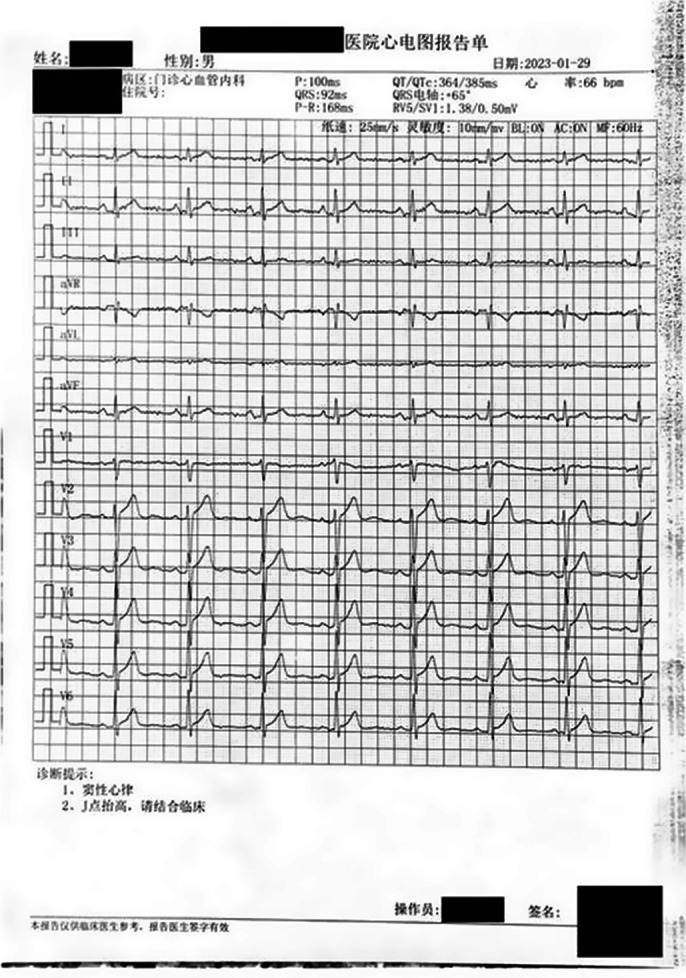

**Image 2:**

**Figure:**
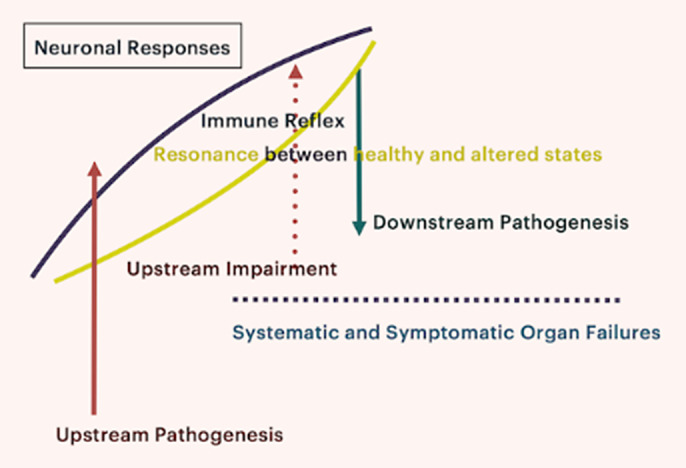

**Image 3:**

**Figure:**
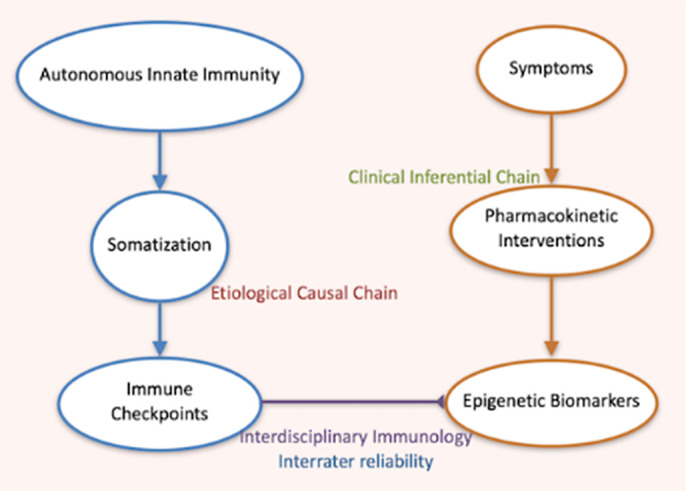

**Conclusions:**

Migraine AEs in NCT05711810 trial is the neuropathic pain resulted from immune activation with initial treatment. Infection depth of neuropathic pains from COVID-19 post-vaccination symptoms is extendable to the presynaptic vesicles with impacts to macrophage activities. Indirect evidences support that SARS-CoV-2 is negative-sensed dsRNA (Pachankis JCMI 2023; 6 1-4).

**Disclosure of Interest:**

None Declared

